# Alterations in skeletal muscle morphology and mechanics in juvenile male Sprague Dawley rats exposed to a high-fat high-sucrose diet

**DOI:** 10.1038/s41598-023-38487-x

**Published:** 2023-07-25

**Authors:** Mauricio Delgado-Bravo, David A. Hart, Raylene A. Reimer, Walter Herzog

**Affiliations:** 1 Human Performance Laboratory, Faculty of Kinesiology, University of Calgary, 2500 University Drive NW, Calgary, AB T2N 1N4 Canada; 2 McCaig Institute for Bone and Joint Health, University of Calgary, Calgary, AB Canada; 3 Department of Biochemistry and Molecular Biology, University of Calgary, Calgary, AB Canada; 4 Department of Surgery, University of Calgary, Calgary, AB Canada; 5 Carrera de Kinesiología, Departamento de Ciencias de la Salud, Facultad de Medicina, Pontificia Universidad Católica de Chile, Santiago, Chile

**Keywords:** Metabolic disorders, Risk factors, Preclinical research

## Abstract

Although once a health concern largely considered in adults, the obesity epidemic is now prevalent in pediatric populations. While detrimental effects on skeletal muscle function have been seen in adulthood, the effects of obesity on skeletal muscle function in childhood is not clearly understood. The purpose of this study was to determine if the consumption of a high-fat high-sucrose (HFS) diet, starting in the post-weaning period, leads to changes in skeletal muscle morphology and mechanics after 14 weeks on the HFS diet. Eighteen 3-week-old male CD-Sprague Dawley rats were randomly assigned to a HFS (C-HFS, n = 10) or standard chow diet (C-CHOW, n = 8). Outcome measures included: weekly energy intake, activity levels, oxygen consumption, body mass, body composition, metabolic profile, serum protein levels, and medial gastrocnemius gene expression, morphology, and mechanics. The main findings from this study were that C-HFS rats: (1) had a greater body mass and percent body fat than control rats; (2) showed early signs of metabolic syndrome; (3) demonstrated potential impairment in muscle remodeling; (4) produced lower relative muscle force; and (5) had a shift in the force–length relationship, indicating that the medial gastrocnemius had shorter muscle fiber lengths compared to those of C-CHOW rats. Based on the results of this study, we conclude that exposure to a HFS diet led to increased body mass, body fat percentage, and early signs of metabolic syndrome, resulting in functional deficits in MG of childhood rats.

## Introduction

Obesity is a global health epidemic of the twenty-first century^1,2^. Approximately 1.4 billion adults (19% of the world’s population) have either overweight or obesity, with the majority residing in westernized societies^3^. Although once a health concern considered more in adulthood, obesity is now prevalent in pediatric populations, with approximately 10% of the Canadian and 19% of the American pediatric population clinically diagnosed as having obesity^2,4,5^. The global obesity epidemic can be largely attributed to a reduction in physical activity, in combination with an increased consumption of modern processed foods^6–8^. Studies commonly report that obesity is detrimental to skeletal muscle health, resulting in a decrease in mobility and the promotion of a sedentary lifestyle^9^, along with changes in skeletal muscle metabolism^10–12^ that can alter fiber typing^13–15^ and increase intramuscular lipid accumulation^10,16,17^. Although a number of human studies have demonstrated that obesity can negatively affect skeletal muscle contractile function^18–20^, findings appear to be inconclusive, as many studies also report that obesity can positively affect skeletal muscle contractile function^3,21–23^. Such contradicting results are particularly the case in pediatric populations, where children with obesity appear to demonstrate an increase^21,22,24^ or no change^25^ in muscle torque production when compared to their lean counterparts.

A major limitation of obesity research is that obesity is typically defined by body anthropometrics (BMI) or composition (% body fat), often neglecting metabolic characteristics^26^. Furthermore, discrepancies in the literature regarding the effects of obesity on skeletal muscle contractile function are often attributed to the potentially confounding effects that diet, body composition, and physical activity may have in the interpretation of findings. This is of particular importance in human research, where it is not feasible to monitor these muscle variables throughout the life span. For this reason, the use of rodent models has become an important tool in the development of our understanding of the effects of obesity on skeletal muscle contractile function. Like human studies, findings from rodent studies are also inconclusive. The majority of evidence from dietary-induced obese rodent models indicates that there is either no change^3,27–32^ or a decrease^33,34^ in maximal force production in isolated skeletal muscle of obese rodents when compared to lean controls. Intriguingly though, similar to that seen in human studies, rodent studies in which the dietary-intervention was implemented in childhood, have found either an increase^31^ or no change in skeletal muscle contractile function^32^ when compared to lean controls.

Based on the inconclusive findings regarding the effects of obesity on skeletal muscle contractile function, it appears important to determine possible metabolic differences amongst the various genetic and dietary rodent models utilized to induce obesity^35–38^. By assessing common markers of metabolic syndrome^39,40^, it may be possible to explain the dichotomy in the literature regarding the effects of obesity on skeletal muscle function^41–44^. We hypothesize that differences in the progression of metabolic syndrome may dictate whether obesity leads to positive or negative changes in skeletal muscle function. Negative muscle adaptations^18–20^, such as a reduction in muscle quality or mass^19^, have been attributed to alterations in skeletal muscle metabolism, suppressing AMPK activity^45^, protein synthesis^46^, and myogenesis^46,47^. Positive muscle adaptations^3,21–23^, such as an increase in muscle volume or cross-sectional area^48^ may be attributed to increased mechanical loads (i.e., greater body mass), which, in combination with an appropriate metabolic profile, may permit muscles to adapt positively^20^.

Energy-dense diets are commonly used to induce obesity in experimental animals. Diets high in saturated fat, simple carbohydrates (e.g., sucrose), or the combination of both, can increase body fat mass, impair glucose tolerance, and trigger inflammation^49,50^. The low-fat control chow diet chosen for this study has been used in many previous studies using Sprague–Dawley rats^51–53^, and thus allows for broad comparison with the literature. The HFS diet is a common obesogenic diet used extensively in our lab^16,54–58^ and has been designed to mimic what is sometimes referred to as a typical western-type diet that is high in sucrose and high in fat content. For the childhood animals used here, slight modifications to our standard HFS diet were made to accommodate the higher protein needs of the growing animals. Previous research suggests that the age at which these diets are introduced to induce obesity plays an important role in the development of metabolic syndrome. For example, male CD-Sprague Dawley rats subjected to a high-fat high-sucrose (HFS) diet in childhood had a milder progression of metabolic syndrome compared to adult rats^58^. This finding may help to explain why obesity induction in childhood may not necessarily lead to negative changes in skeletal muscle function^32^. The purpose of the present study was to monitor the primary factors contributing to obesity (i.e., diet, physical activity, body mass) during childhood/adolescence, and to determine if skeletal muscles of male CD-Sprague Dawley rats subjected to a HFS diet in childhood show signs of degeneration when subsequently assessed as adults. We hypothesized that despite the early progression of metabolic syndrome, the consumption of a HFS diet in childhood would not have a detrimental effect on medial gastrocnemius muscle morphology and mechanics. With an ever-increasing proportion of obesity in childhood, this study will add potentially important information regarding the relationship between early childhood obesity and muscle function and muscle health during growth and maturation.

## Methods

### Rats and dietary intervention

Starting at 3 weeks of age, eighteen male Sprague–Dawley rats were randomly assigned to either a high-fat high-sucrose diet (C-HFS; n = 10) or a standard chow control diet (C-CHOW; n = 8) for 14-weeks. All animals were provided food and water ad libitum. The control diet was standard rodent chow (Lab Diet 5001, St. Louis, MO, USA) consisting of 5% of total weight as fat, 47.5% carbohydrates (only 4% from sucrose), 25% protein, 12.5% from fiber and micronutrients, and 10% moisture. The HFS diet (Dyets, Bethlehem, PA, USA) consisted of 20% of total weight as fat, 50% sucrose, 20% protein, and 10% from fiber and micronutrients. From 3 to 10 weeks of age, the diet had slightly higher protein content to meet the requirements for growth (20% of total weight as fat, 46% sucrose, 24% protein, and 10% from fiber and micronutrients. All experimental protocols and procedures were approved by the University of Calgary’s Life and Environmental Sciences Animal Care Committee (Protocol #: AC16-0130), and all methods were performed in accordance with guidelines and regulations at the University of Calgary, as well as complying with ARRIVE guidelines^59^ for the reporting of animal experiments.

### Weekly growth characteristics

Each week, body mass was recorded, and animals were individually placed in a metabolic chamber (Oxymax, Columbus Instruments, OH, USA) for 24-h to track energy intake, activity level, and oxygen consumption over the 14-week intervention period. Energy intake was calculated based on food consumption and expressed in kcal/day. Activity level was based on the average number of infrared beam breaks per 10-min interval. Oxygen consumption was calculated based on O_2_ kinetics (VO_2_ = V_i_ O_2i_ − V_o_ O_2o_) and expressed as milliliters per minute relative to body mass (ml/min/kg).

### Body composition

At the end of the feeding period, rats were anaesthetized with isoflurane, and body composition was analyzed via dual x-ray absorptiometry (DXA) using software for small animals (Hologic QDR 4500; Hologic, Bedford, MA, USA). Three scans were performed per animal, with the means being used for analyses.

### Markers of metabolic syndrome

Secondary analyses were performed on metabolic markers collected from previously published data^58^ to make direct comparisons between C-CHOW and C-HFS rats. Briefly, at the end of the 14-week intervention period, and following a 16 h fast, rats were anesthetized with isoflurane, blood was drawn, and the following analyses were conducted for glucose, insulin, lipid, and cholesterol determination. To assess glucose response, an oral glucose tolerance test (OGTT) was performed with a 2 g/kg body weight glucose load to assess dynamic blood glucose (Glucose-OGTT) from 0 to 120 min. To assess whole body insulin sensitivity (Insulin-OGTT)^60^, the composite insulin sensitivity index (CISI) was determined using proxy measures from the OGTT^61^. Standard colorimetric assays (Calgary Lab Services, Calgary, AB, Canada) were used to analyze fasting serum triglycerides (TG), high-density lipoprotein cholesterol (HDL-C), and total cholesterol (Total-C). LDL cholesterol (LDL-C) was calculated as: Total-C minus HDL-C minus (TG/2.2).

### Serum hormones, growth factors and cytokines

A Rat 27 Multiplex Discovery Assay with Luminex® xMAP technology (Eve Technologies, AB, Canada) was utilized to assess 27-markers in the serum (Eotaxin, EGF, Fractalkine, IL-1α, IL-1β, IL-2, IL-4, IL-5, IL-6, IL-10, IL-12 (p70), IL-13, IL-17A, IL-18, IP-10/CXCL10, GRO/KC, IFN-γ, TNF-α, G-CSF, GM-CSF, MCP-1, leptin, LIX, MIP-1α, MIP-2, RANTES, VEGF) according to the manufacturers specifications. Analyte values that were outside of the curve or out of range (OOR) were adjusted accordingly, based on their assigned low fluorescence intensity (FI) value. Two analytes (GRO/KC, IFN-γ) were removed from the analyses, as many values were out of range.

### Medial gastrocnemius gene expression and morphology

#### Tissue preparation

Following mechanical testing, animals were euthanized, and the left medial gastrocnemius was harvested. Medial gastrocnemius wet weight was measured, the muscle was snap frozen in isopentane, immersed in liquid nitrogen, and stored at − 80 °C.

#### Gene expression

Reverse transcription quantitative polymerase chain reaction (rt-qPCR) was used to assess gene expression for a variety of cellular markers in the medial gastrocnemius muscle. Markers assessed those related to cellular stress (IL-6, MCP-1, TNFα), lipid droplet storage (Fsp27, Leptin, Adiponectin), cell death (IL-1β, Cytochrome-C, Caspase-3), and myogenesis (Pax7, MyoD, Myf5, Myogenin, Mrf4). Frozen tissue samples were powdered at − 80 °C in liquid nitrogen with a Braun mikro-dismembrator (Braun Biotech International, Allentown, PA) with total RNA being isolated using the TriSpin method^62^. qPCR analyses were performed using an iCycler (BioRad Laboratories, Inc., Mississauga, ON), with all analyses performed in duplicate on a single 96 well plate, using the 2^-∆∆CT^ method, under optimal conditions that conformed to qPCR criteria. 18S rRNA was used as the housekeeping gene to normalize gene expression values across samples. Sequences for all validated rat specific primers used are provided in the Supplementary Material—Table S1.

#### Collagen content

12 µm cross-sections of medial gastrocnemius tissue samples were prepared on a cryostat and mounted on slides. Slides were air dried and fixed in 10% Neutral Buffered Formalin for 7 days. Slides were immersed in Bouin’s fixative solution at 60 °C for 1 h, washed with distilled water, and placed at room temperature for 1 h in PicroSirius Red Solution (Sigma-Aldrich, Oakville, ON, Canada). Slides underwent two quick washes in 1% acetic acid, dehydrated in 3 quick changes of 100% ethanol, cleared with 2 × 1 min in xylene, and mounted with DPX mounting medium. Stained muscle cross-sections were imaged at 10 × magnification on an Olympus BX53 light microscope (Olympus, Center Valley, PA, USA) and analyzed using a custom MatLab program. The entire muscle cross-section was captured, and collagen content was calculated as a percentage of the muscle cross-sectional area.

#### Lipid content

Medial gastrocnemius tissue samples (~ 30 mg) were freeze-dried and ground into a powder. KOH solution was added to tissue samples and placed in an oven at 70 °C for 1-h, and then left to sit at room temperature overnight. 2 M Tris–HCL (pH 7.5) was added, tubes were centrifuged for 5 min, and the supernatant was collected. 200 μl of GPO triglyceride reagent (Cat #T7532, Pointe Scientific Inc., Lincoln Park, MI, USA) was added to each well of a 96-well plate and pre-warmed at 37 °C. Standards (Cat #T7531-STD, Pointe Scientific Inc., Lincoln Park, MI, USA) and samples were then added to each well, and the 96-well plate was warmed at 37 °C for another 5 min. A SpectraMax 190 spectrophotometer (Molecular Devices, San Jose, CA, USA) was used to quantify the intensity of light at a 500 nm wavelength for each sample. Using 6 standards (0–32 µg), a linear regression was formulated, and triglycerides content was determined for each sample. Medial gastrocnemius lipid content was expressed as a percentage of muscle wet weight.

### Medial gastrocnemius muscle mechanics

#### Mechanical testing protocol

The medial gastrocnemius of the right hind-limb was exposed, and its distal end released with a piece of calcaneal bone. A nerve cuff electrode was secured around the exposed tibial nerve, permitting electrical stimulation of the medial gastrocnemius. Animals were positioned prone in a hammock and secured in a stereotactic frame, with the hind limb securely fixed with bone pins. The medial gastrocnemius tendon was attached to a force transducer (LCM201-100N, OMEGA Engineering Inc., Stamford, CT, USA) via a bone clamp. The force transducer was mounted to a servomotor (404LXR, Parker Hannifin Corporation, Irwin, PA, USA) operated by Motion Planner Software (Motion Planner 16, Parker Hannifin Corporation, Irwin, PA, USA). The nerve cuff electrode was connected to a Grass S8800 stimulator (Grass Technologies, West Warwick, RI, USA) and medial gastrocnemius force and length were recorded and displayed using WinDaq software (DATAQ Instruments, Akron, OH, USA). Core body temperature (~ 37 °C) was closely monitored, and the exposed MG was covered with warm saline-soaked gauze and warmed under a heat lamp.

#### Muscle force–length relationship (FLR)

The medial gastrocnemius was set to its minimum in vivo muscle length (C-CHOW: 35 ± 0.6 mm; C-HFS: 35 ± 0.5 mm), defined as 0 mm. For each trial, the MG was stimulated supra-maximally so that all motor units were activated^63^. Maximal isometric force was then determined from this initial length at 1 mm increments. Once stretched to its new length, the medial gastrocnemius was held for 5 s, allowing passive force to relax. The average medial gastrocnemius passive force was recorded over 500 ms prior to muscle stimulation. The muscle was then activated using fused tetanic contractions (Duration: 200 ms, Frequency: 120 Hz, Pulse duration: 0.1 ms). Following each activation, the medial gastrocnemius was returned to its minimum in vivo length (0 mm) for 3 min. Total force was defined as the peak force produced by the medial gastrocnemius during activation. Medial gastrocnemius active force was calculated as the total force minus the passive force at the corresponding lengths, realizing that this may slightly underestimate the active force^64^. Medial gastrocnemius optimal length was defined as the muscle length at which medial gastrocnemius produced the maximal active isometric force^65^.

#### Muscle maximal force

Absolute muscle force was defined as the maximal active isometric force produced at optimal length. Muscle quality was defined as absolute muscle force normalized to muscle mass. Muscle stress was defined as absolute muscle force normalized to physiological cross-sectional area (PCSA), assessed via histological cross-sections. Relative muscle force was defined as absolute muscle force normalized to body mass.

### Statistical analysis

All statistical analyses were run using SPSS software (IBM SPSS Statistics 23, Chicago, IL, USA). Data were assessed for outliers using box plots, normality using the Shapiro–Wilk’s test, and equality of variance using Mauchly’s test of sphericity (Greenhouse–Geisser correction) and Levene’s test for homogeneity of variance. A two-way repeated measures ANOVA was performed to determine any significant interactions between Diet × Time on weekly caloric intake, body mass, activity level, and oxygen consumption over the 14-week intervention period. A two-way mixed ANOVA was used to determine any significant interactions between Diet × Length of medial gastrocnemius force for the active and passive FLR. If a significant two-way interaction was found, pairwise comparisons were reported using Bonferroni post hoc adjustments. Independent t-tests were conducted to determine differences in body composition, markers of metabolic syndrome, serum proteins (27-plex), and medial gastrocnemius gene expression, morphology, and mechanics between C-CHOW and C-HFS rats. If data were not normally distributed, Mann–Whitney U test were run. All data are presented in the Tables and Supplementary Tables as means ± 1 standard deviation. Data in Figures represent means, with error bars representing standard deviations. Data were considered statistically significant at p < 0.05, two-tailed.

## Results

### Energy intake

**Figure 1:**
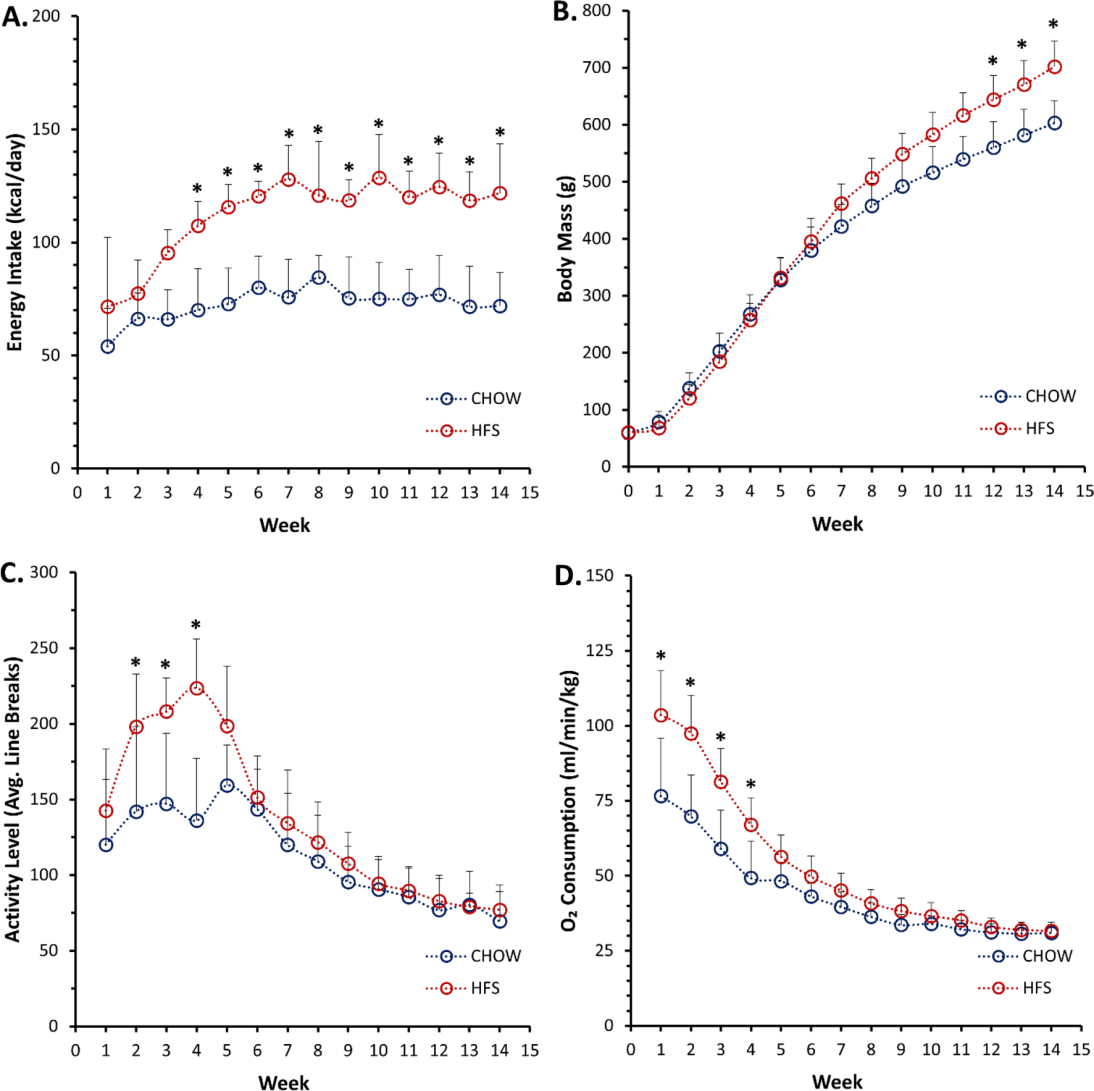
Weekly animal characteristics over feeding period with the chow diet or the high fat high sugar (HFS) Diet. (**A**) Displays energy-intake tracked over 24-h. (**B**) Displays body mass. (**C**) Displays average activity levels per 10-min window, tracked over 24-h. (**D**) Displays O_2_ consumption per min, per kg of body mass, tracked over a 24-h. X-axis represents duration of the feeding period in weeks. All the Diet × Time interactions was significant (p < 0.05). Data points represent the average, with error bars representing the standard deviation. “*” represents a statistically significant (p < 0.05) between-group difference.

There was a statistically significant main effect of Time (p < 0.001), Diet (p < 0.001), and Diet × Time interaction for weekly caloric intake (p = 0.002). Based on post hoc comparison, C-HFS rats exhibited a significant increase in daily energy intake from weeks 4–14 of the feeding intervention period when compared to C-CHOW rats, ranging from a 44% greater energy intake at week-4 (p < 0.001), to a 71% greater energy intake at week-14 (p = 0.001, Fig. 1A). Over the duration of the intervention period, on average, C-HFS rats (112 ± 3 kcal/day) consumed 37 kcal/day (29 to 50 kcal/day) more than the C-CHOW rats (75 ± 3 kcal/day). Given that 39.2% of total calories in the C-HFS diet is provided by fat, C-HFS rats consumed on average 43.9 kcal/day as fat.

### Body mass

Regarding weekly body mass assessments, there was a statistically significant main effect of Time (p < 0.001), Diet (p = 0.029), and Diet × Time interaction (p < 0.001). Post hoc analysis showed that the body mass of the HFS fed rats was greater from week-12 to the end of the intervention period when compared to C-CHOW rats (Fig. 1B). At the end of the intervention period, the body mass of C-HFS rats was 16% greater than that of the C-CHOW rats (p = 0.001, Table 1).

**Table 1:** Body composition.

Body composition	Units	CHOW	HFS	Effect size	Significance
		Mean ± SD	Mean ± SD	Cohen's d	p-value
Body mass	g	**603 ± 38**	**701 ± 45**	**2.35**	**p = 0.001**
Lean mass	g	498 ± 18	473 ± 29	0.99	p = 0.058
Fat mass	g	**104 ± 44**	**227 ± 44**	**2.75**	**p = 0.001**
Body fat	%	**17 ± 5**	**32 ± 4**	**2.99**	**p = 0.001**
BMD	g/cm^2^	0.16 ± 0.01	0.17 ± 0.01	1.00	p = 0.161

Data represent body composition assessed using DXA. Bold numbers indicate a statistically significant difference (p < 0.05).

*BMD* bone mineral density.

### Activity levels

There was a statistically significant main effect of Time (p < 0.001), Diet (p = 0.015), and Diet × Time interaction (p < 0.001). C-HFS rats had up to 64% greater activity levels when compared to C-CHOW rats (week-4, p = 0.001), with activity levels being significantly greater between weeks 2–4 of the intervention period (Fig. 1C). These increased activity levels were accompanied by a significant main effect of Time (p < 0.001), Diet (p = 0.004), and Diet × Time interaction for oxygen consumption (p < 0.001), with up to 40% greater oxygen consumption in C-HFS rats than C-CHOW rats (week-2, p < 0.001), and C-HFS rats having significantly greater oxygen consumption between weeks 1–4 of the intervention period (Fig. 1D).

### Body composition

**Figure 2:**
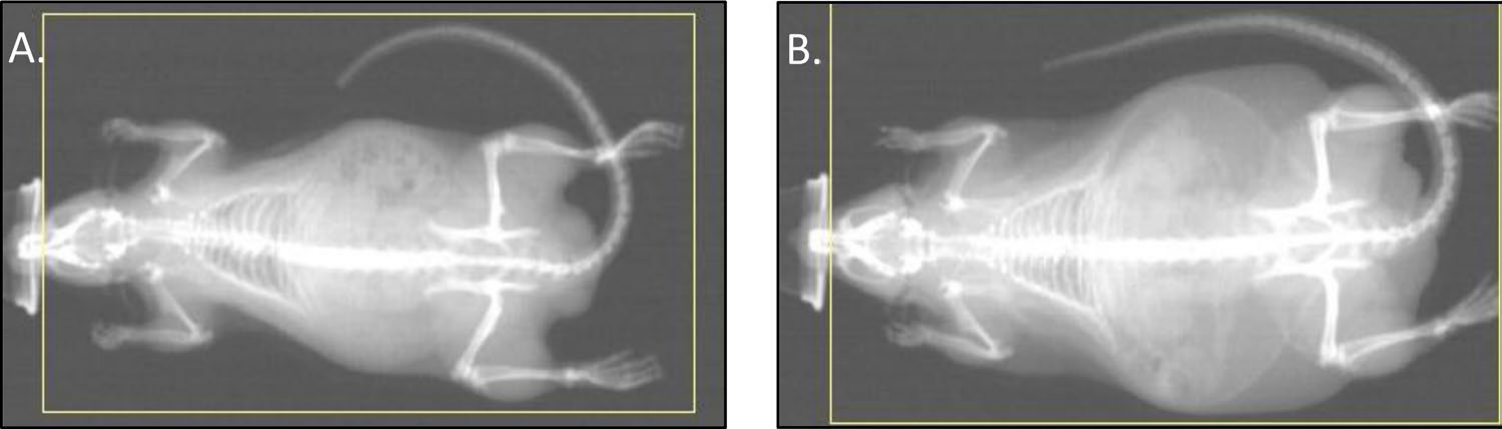
Body composition. Body composition was analyzed via dual x-ray absorptiometry (DXA). Images display sample scans for (**A**) CHOW and (**B**) HFS rats (after 14-weeks of diet).

At the end of the intervention period, C-HFS rats had a mean 117% greater fat mass (p = 0.001) and an 88% greater percent body fat content (p = 0.001) than the C-CHOW control rats (Fig. 2). C-HFS rats also had 5% less lean body mass than the C-CHOW rats, which approached statistical significance (p = 0.058). There were no significant differences in bone mineral density (BMD) between the two groups of animals (p = 0.161, Table 1).

### Markers of metabolic syndrome

There were no differences in fasting blood glucose and insulin levels between the C-HFS and the C-CHOW group rats. No differences were detected in glucose area under the curve (AUC) during the OGTT. Insulin AUC, however, was 113% greater in C-HFS rats compared to that seen in the C-CHOW rats (p = 0.027), resulting in a 54% decrease in insulin sensitivity in C-HFS rats (p = 0.019, Table 2). The time course of insulin and glucose response during the OGTT is included in Supplementary Material—Table S2 and Fig. S1. C-HFS rats did not exhibit elevated TG levels when compared to C-CHOW rats, but cholesterol levels (LDL-C: 154%, HDL-C: 46%, Total-C: 42%) were significantly elevated. However, there were no detectable differences in the TG:HDL ratio (p = 0.919, Table 2).

**Table 2:** Markers of metabolic syndrome.

Metabolic syndrome	Units	CHOW	HFS	Effect size	Significance
		Mean ± SD	Mean ± SD	Cohen's d	p-value
Fasting Glucose	mmol/L	5.7 ± 0.7	5.9 ± 0.5	0.33	p = 0.538
Fasting Insulin	pmol/L	134 ± 54	191.1 ± 75.8	0.85	p = 0.110
Glucose-OGTT	AUC	1286 ± 210	1470.0 ± 243.1	0.81	p = 0.130
Insulin-OGTT	AUC	**20,224 ± 6917**	**43,121.0 ± 23,113.6**	**1.34**	**p = 0.027**
Insulin Sensitivity	CISI	**1.00 ± 0.47**	**0.46 ± 0.33**	**− 1.33**	**p = 0.019**
Triglycerides	mg/dl	107 ± 25	132.52 ± 54.64	0.59	p = 0.263
LDL-C	mg/dl	**13 ± 10**	**35.24 ± 16.35**	**1.55**	**p = 0.008**
HDL-C	mg/dl	**52 ± 10**	**76.03 ± 12.05**	**2.13**	**p = 0.001**
Total-C	mg/dl	**68 ± 12**	**96.77 ± 9.77**	**2.55**	**p = 0.001**
TG:HDL-C	Ratio	1.91 ± 0.22	1.87 ± 1.06	− 0.05	p = 0.919

Fasting blood glucose, insulin, lipid, and cholesterol levels were taken following a 16-h fast. Oral glucose tolerance tests (OGTT) were administered to assess the dynamic glucose (Glucose-OGTT) and insulin (Insulin-OGTT) response based on area under the curve (AUC). Whole body insulin sensitivity was determined using the composite insulin sensitivity index (CISI). Statistically significant differences are highlighted in bold.

*TG* triglycerides, *LDL-C* low-density lipoprotein cholesterol, *HDL-C* high-density lipoprotein cholesterol, *Total-C* total cholesterol.

### Serum hormones, growth factors and cytokine levels

C-HFS rats had 1.5-fold-greater leptin levels (p = 0.001), 1.5-fold-lower MIP-2 levels (p = 0.039), and 1.6-fold-lower IL-6 levels (p = 0.006) when compared to C-CHOW rats (Table 3). There was also a trend (p = 0.054) for VEGF to be higher in C-HFS versus C-CHOW animals.

**Table 3:** Serum hormones, growth factors, & cytokines.

Serum markers	Units	CHOW	HFS	Effect size	Significance
	pg/ml	Mean ± SD	Mean ± SD	Cohen's d	p-value
Satiety	Leptin	**33,642 ± 16,714**	**48,838 ± 5137**	**1.23**	**p = 0.001**
Mitosis (proliferation/differentiation)	G-CSF	15 ± 16	3 ± 5	− 0.98	p = 0.130
	GM-CSF	41 ± 19	38 ± 17	− 0.19	p = 0.705
	EGF	239 ± 212	189 ± 99	− 0.30	p = 0.557
	VEGF	22 ± 11	46 ± 28	1.10	p = 0.054
Immune cell recruitment	LIX	2712 ± 515	2756 ± 455	0.09	p = 0.859
	MIP-1α	18. ± 6.5	16 ± 2	− 0.23	p = 0.654
	MCP-1	660 ± 113	623 ± 229	− 0.20	p = 0.691
	Fractalkine	49 ± 13	49 ± 9	− 0.06	p = 0.909
	Eotaxin	9 ± 1	8 ± 2	− 0.33	p = 0.518
	RANTES	900 ± 343	781 ± 167	− 0.44	p = 0.394
	IP-10	309 ± 43	300 ± 47	− 0.21	p = 0.678
	MIP-2	**52 ± 14**	**33 ± 17**	**− 1.14**	**p = 0.039**
Innate inflammatory response	IL-1α	105 ± 74	56 ± 30	− 0.86	p = 0.107
	IL-1β	144 ± 111	98 ± 34	− 0.56	p = 0.878
	TNFα	7 ± 5	5 ± 2	− 0.55	p = 0.505
Th1 cells	IL-12 (p70)	184 ± 47	194 ± 90	0.14	p = 0.782
	IL-18	689 ± 230	808 ± 161	0.60	p = 0.250
	IL-2	115 ± 47	96 ± 36	− 0.43	p = 0.400
Th2 cells/ B cells	IL-4	24 ± 2	19 ± 6	− 1.06	p = 0.065
	IL-5	52 ± 12	51 ± 11	− 0.04	p = 0.935
	IL-13	16 ± 7	11 ± 4	− 0.85	p = 0.110
Treg	IL-10	102 ± 76	62 ± 31	− 0.68	p = 0.442
Th17 cells	IL-6	**1090 ± 259**	**702 ± 212**	**− 1.64**	**p = 0.006**
	IL-17α	17 ± 10	11 ± 5	− 0.70	p = 0.182

Serum hormones, growth factors, and cytokines were analyzed following a 16-h fast using a 27-plex assay to assess extracellular protein content. GRO-KC and IFNγ were removed due to many values being out of range. Bold- indicates a statistically significant difference (p < 0.05).

### Medial gastrocnemius gene expression

C-HFS rats exhibited significant alterations in the medial gastrocnemius gene expression compared to the C-CHOW animals, predominantly in myogenic and pro-inflammatory markers (Table 4). C-HFS rats had a 1.3-fold-greater gene expression for IL-1β (p = 0.002), a 2.0-fold-greater gene expression for Caspase-3 (p = 0.001), and a 1.5-fold-greater gene expression for TNFα, which approached statistical significance (p = 0.053) when compared to C-CHOW rats (Table 4). C-HFS rats had a 2.2 to 3.0-fold-greater gene expression of the myogenic markers (MyoD, Myf5, Myogen) when compared to C-CHOW rats (Table 4).

**Table 4:** Medial gastrocnemius gene expression.

Medial gastrocnemius	Gene (normalized to 18S)	CHOW Mean ± SD	HFS Mean ± SD	Effect size Cohen's d	p-value
Cellular stress	IL-6	0.03 ± 0.03	0.05 ± 0.02	0.78	p = 0.176
	MCP-1	0.01 ± 0.01	0.02 ± 0.01	1.00	p = 0.316
	TNFα	0.12 ± 0.08	0.18 ± 0.06	0.85	p = 0.053
Lipid droplet	Fsp27	0.76 ± 0.44	1.05 ± 0.66	0.52	p = 0.299
	Leptin	0.26 ± 0.21	0.28 ± 0.10	0.12	p = 0.780
	Adiponectin	1.06 ± 0.50	1.03 ± 0.35	0.07	p = 0.876
Cell death	IL-1β	**0.03 ± 0.01**	**0.04 ± 0.01**	**1.00**	**p = 0.002**
	Cyto-C	1.58 ± 0.48	1.73 ± 0.22	0.40	p = 0.396
	Caspase-3	**0.62 ± 0.25**	**1.25 ± 0.33**	**2.15**	**p = 0.001**
Muscle atrophy	MAF	1.27 ± 0.89	1.00 ± 0.25	0.41	p = 0.416
	MURF	1.06 ± 0.70	0.81 ± 0.32	0.46	p = 0.369
Myogenesis	Pax7	0.68 ± 0.23	0.71 ± 0.38	0.10	p = 0.828
	MyoD	**0.42 ± 0.20**	**1.26 ± 0.55**	**2.03**	**p = 0.001**
	Myf5	**0.30 ± 0.16**	**0.66 ± 0.44**	**1.09**	**p = 0.038**
	Myogenin	**0.29 ± 0.11**	**0.71 ± 0.18**	**2.82**	**p = 0.001**
	MRF4	0.84 ± 0.45	1.16 ± 0.46	0.70	p = 0.162

Data represent medial gastrocnemius gene expression normalized to 18S rRNA, assessed via qPCR. Bold indicates a statistically significant difference (p < 0.05).

### Medial gastrocnemius morphology

**Figure 3:**
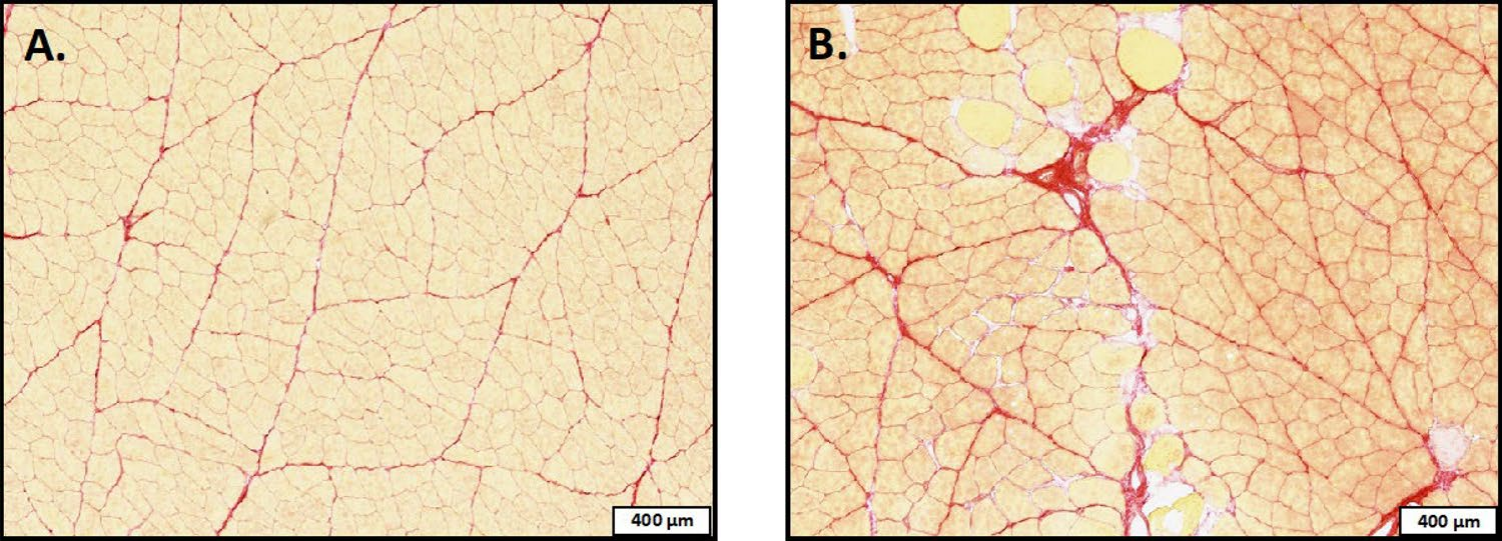
Medial gastrocnemius muscle morphology. Images display histological cross-sections of the medial gastrocnemius muscle stain with Picro-Sirius Red for (**A**) an exemplar C-CHOW and (**B**) an exemplar C-HFS group rat. Tissue was obtained after sacrifice. Muscle fibers are stained in yellow, and collagen is stained in red. Scale bar represents 400 µm.

There were no significant differences detected regarding medial gastrocnemius muscle mass (p = 0.448), PCSA (p = 0.211), or lipid content (p = 0.658) between the two groups of rats (Table 5). The only significant difference in muscle morphology was in collagen content (Fig. 3), with C-HFS rats having a mean 58% greater percent collagen content (p = 0.004) than the C-CHOW rats (Table 5).

**Table 5:** Medial gastrocnemius muscle morphology & mechanics.

Medial gastrocnemius	Units	CHOW	HFS	Effect size	Significance
		Mean ± SD	Mean ± SD	Cohen's d	p-value
Mass	g	1.98 ± 0.17	1.91 ± 0.21	0.37	p = 0.448
Volume	mm^3^	1798 ± 197	1864 ± 163	0.37	p = 0.448
PCSA	mm^2^	66.75 ± 9.05	71.14 ± 5.08	0.60	p = 0.211
Contractile PCSA	mm^2^	62.11 ± 8.76	63.35 ± 5.81	0.17	p = 0.722
Collagen content	%	**7.00 ± 1.84**	**11.04 ± 2.91**	**1.66**	**p = 0.004**
Lipid content	%	0.84 ± 0.20	0.89 ± 0.25	0.22	p = 0.658
Absolute force	N	14.60 ± 1.76	13.93 ± 2.43	0.32	p = 0.524
Muscle stress	N/mm^2^	0.22 ± 0.06	0.20 ± 0.03	0.42	p = 0.286
Muscle quality	N/g	7.73 ± 1.16	7.06 ± 1.13	0.59	p = 0.233
Relative force	N/kg	**24.34 ± 3.55**	**19.92 ± 3.64**	**1.23**	**p = 0.020**

Data represents medial gastrocnemius (MG) muscle morphology and mechanics in an *in-situ* muscle preparation. PCSA—physiological cross-sectional area. Contractile PCSA—PCSA minus collagen content. Absolute Force- maximal isometric force. Muscle Quality—maximal force normalized to MG mass. Muscle Stress—maximal force normalized to physiological cross-sectional area (PCSA). Relative Force—maximal force normalized to body mass. Bold- indicates a statistically significant difference (p < 0.05).

### Medial gastrocnemius maximal force

With no differences in medial gastrocnemius muscle mass (p = 0.448), PCSA (p = 0.211, Table 5), or absolute muscle force (p = 0.524), no differences in muscle quality (p = 0.233) and muscle stress (p = 0.286, Table 5) were detected between C-CHOW and C-HFS rats. The only significant difference was that the relative muscle force was 18% lower in C-HFS rats than the C-CHOW rats (p = 0.020, Table 5).

### Medial gastrocnemius force length relationship: (FLR)

**Figure 4:**
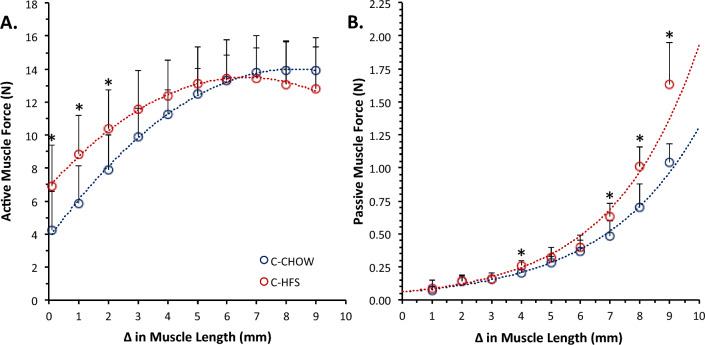
Medial gastrocnemius- active and passive force length relationships. Medial gastrocnemius change in muscle length is displayed on the x-axis. 0 mm represents the minimum in-vivo muscle length (C-CHOW: 35 ± 0.6 mm; C-HFS: 35 ± 0.5 mm). (**A**) Active force is displayed on the y-axis with data points representing average isometric force. Data fitted with polynomial trend lines, with C-CHOW having a R^2^ = 0.99 and C-HFS having a R^2^ = 0.99. (**B**) Passive force displayed on the y-axis with data points representing average passive force. Data fitted with exponential trend lines, with C-CHOW having a R^2^ = 0.98 and C-HFS having a R^2^ = 0.98. Error bars represent standard deviations. “*” represents a statistically significant (p < 0.05) between-group difference.

Despite similar tibia lengths (C-CHOW: 44.4 ± 1.3 mm; C-HFS: 45.6 ± 1.3 mm), and the medial gastrocnemius demonstrating similar minimum in-vivo lengths (C-CHOW: 35 ± 0.6 mm; C-HFS: 35 ± 0.5 mm) and absolute muscle forces (Table 5), the FLR of the C-HFS rats was shifted towards shorter muscle fiber lengths for the active (Fig. 4A) and passive forces (Fig. 4B) compared to the FLR of the C-CHOW rats. For the C-HFS rats, maximal active force occurred at a shorter average muscle length than in the C-CHOW rats (CHOW: 8.6 mm, HFS: 6.9 mm, Fig. 4A). Because of this shift, C-HFS rats produced 32-to-63% greater active force at short muscle lengths (0–2 mm) when compared to the C-CHOW rats. C-HFS rats also produced 30-to-57% greater medial gastrocnemius passive forces at long muscle lengths (4 mm, 7–9 mm) when compared to the passive forces for the C-CHOW rats (Fig. 4B).

## Discussion

The purpose of this study was to determine if the consumption of a HFS diet, starting at weaning, leads to alterations in skeletal muscle morphology and mechanics in male Sprague Dawley rats, when compared to animals fed a CHOW diet. The main findings from this study were that C-HFS rats: (1) had a greater body mass and percent body fat; (2) showed early signs of metabolic syndrome (i.e. elevated LDL cholesterol and reduced insulin sensitivity); (3) demonstrated potential impairment in muscle remodeling; (4) produced lower relative muscle force; and (5) had a shift in the FLR, indicating that the medial gastrocnemius operated at shorter muscle fiber lengths when compared to C-CHOW rats.

In monitoring the main contributors to obesity (i.e., intake of an energy dense diet, activity levels) over childhood and adolescence in Sprague–Dawley rats, we found that consumption of a HFS versus CHOW diet resulted in an increased total body mass, fat mass, and percent body fat, similar to reports in adult rats^54,55^. Intriguingly, despite a significant increase in energy intake in C-HFS rats from week 4 onwards, a significant difference in body mass only occurred starting in week-7 of the feeding intervention period, approximately when rats enter puberty and young adulthood^54^. Whether there is a potential role for sex hormones in the response to diet remains to be determined.

The delayed increase in body mass may be attributed to the greater activity levels that were observed at the beginning of the HFS-diet intervention period in the C-HFS group rats compared to the C-CHOW group rats. A leptin stimulated increase in sympathetic nervous system activity^44^ may explain this increase in activity levels in the C-HFS group rats. Elevated blood leptin levels^66^ have been shown to stimulate an increase in skeletal muscle metabolism^67^, as a means to help maintain metabolic homeostasis^68,69^. This pathway may also help to explain the increase in oxygen consumption seen in C-HFS rats compared to C-CHOW rats. Surprisingly, despite an increase in activity levels and body mass, C-HFS rats had a 5% lower lean body mass (p = 0.058) than C-CHOW rats, an indication of potential impairments in muscle regeneration in C-HFS rats^46,70–72^.

With the energy overload of the HFS diet placing significant metabolic stress on adipose tissues of C-HFS rats, as indicated by a decrease in insulin sensitivity^73^ and elevated blood leptin levels^74^, it was surprising to observe that both blood glucose and triglyceride levels were similar in C-HFS and C-CHOW group rats. This being said, C-HFS rats required elevated insulin secretion in order to appropriately regulate systemic glucose^75^. This display of resistance to the actions of insulin is reflected in the significantly lower CISI value in C-HFS rats, confirming their reduced insulin sensitivity compared to C-CHOW. Despite higher total-C, LDL-C and HDL-C in C-HFS rats, the similar TG:HDL-C ratios of the C-HFS and C-CHOW rats suggests that elevated blood cholesterol levels in the C-HFS rats may not be harmful to metabolic health yet but could progress if the rats remained on the diet for a longer period of time^76^. The ability of adipose tissue in C-HFS rats to buffer the energy overload of the HFS diet is likely what allowed C-HFS rats to maintain metabolic homeostasis^77^, mitigate the presence of a chronic low-grade inflammatory state^78^, and prevent the spillover and ectopic storage of lipids in skeletal muscle^44,79^. Therefore, unlike the 22% lipid content previously reported in the vastus lateralis of adult rats consuming a HFS diets^16^, C-HFS rats only showed 1% lipid content in the medial gastrocnemius, similar to that seen in the C-CHOW rats. There were no detectable differences in medial gastrocnemius lipid content between C-HFS and C-CHOW. The two groups had similar levels of Fsp27, leptin, and adiponectin gene expression, suggesting that lipid droplets were not expanding^80^ and that muscle glucose and fatty acid metabolism^44,81^ were unaltered. The biggest difference in muscle morphology was the 58% greater collagen deposition in the medial gastrocnemius of C-HFS rats when compared to C-CHOW rats, providing further indication of potential impairments in muscle regeneration, perhaps via fibrosis, in the C-HFS rats^46,70–72^.

**Figure 5:**
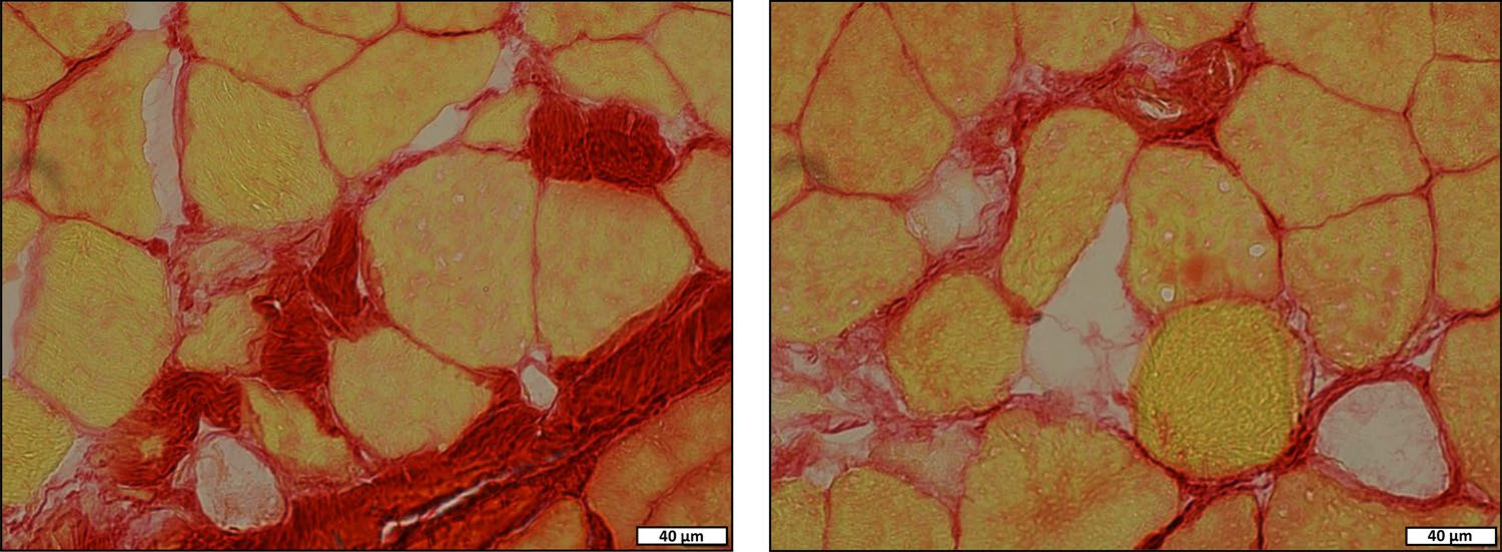
Indication of Impaired Muscle Regeneration in HFS rats. Images display exemplar histological cross-sections of the medial gastrocnemius muscle stained with Picro-Sirius Red. Muscle fibers are stained in yellow and collagen is stained in red. Scale bar represents 40 µm.

Impairments in muscle regeneration, particularly if sustained over a long period of time, is a hallmark in the etiology of many degenerative muscular diseases that reduce muscle contractile function^82^. Based on the elevated levels of TNFα, IL-1β, and caspase-3 gene expression in the medial gastrocnemius of C-HFS rats, our findings indicate elevated levels of muscle tissue degeneration in C-HFS rats compared to C-CHOW rats^71^. Increased IL-1β levels are associated with numerous degenerative diseases^83,84^ due to IL-1β’s ability to initiate potent inflammatory responses^85^. Furthermore, IL-1β and caspase-3 (i.e., executioner caspase) are known to play important roles in apoptosis^71,86^. Elevated TNFα may have initiated the pro-inflammatory response in the medial gastrocnemius of C-HFS rats, as TNFα is known to stimulate IL-1β secretion by phagocytes, along with activating caspase-3 via caspase-8 extrinsic apoptosis pathways^86,87^. Intriguingly, despite elevated levels of muscle tissue degeneration in C-HFS rats, and contrary to previous reports in the obesity literature^46,47^, C-HFS rats had elevated, rather than reduced, gene expression levels for myogenic markers in the medial gastrocnemius when compared to C-CHOW rats^71,88^. This combination of elevated muscle degeneration with a simultaneous increase of markers for muscle regeneration in C-HFS rats, indicates that significant remodeling was likely occurring in C-HFS rats compared to C-CHOW rats^71^. With the overproduction of extracellular matrix components associated with fibrotic events, as observed in the medial gastrocnemius of C-HFS rats (Fig. 5) and known to eventually lead to loss in muscle function^71,89^, these findings further suggest that C-HFS rats demonstrated impairments in muscle regeneration^43,70,72^.

However, the increased collagen deposition in C-HFS rats did not affect the number of sarcomeres-in-parallel in the medial gastrocnemius, resulting in the same force capacity in C-HFS and C-CHOW group rats, in agreement with previous studies^27,32^. The increased collagen deposition in C-HFS compared to C-CHOW rats did not alter medial gastrocnemius PCSA, thus resulting in similar muscle stresses (i.e., Force / PCSA) in the two groups of animals. This latter finding contradicts previous obesity studies in rodents^31,33,34^, but they were performed on a different muscle (extensor digitorum longus and soleus). With force, muscle mass, and muscle quality the same between C-HFS and C-CHOW group rats, our results are contrary to what has been reported in other studies of obese rodents^31^. The reduction in a lower relative muscle force (i.e., force / body mass) in C-HFS compared to C-CHOW group rats is entirely attributable to the increased body mass, and this result agrees with previous observations in rodents^28,33^. Medial gastrocnemius muscle force was not significantly altered in C-HFS compared to C-CHOW group rats. Whether this is beneficial or detrimental is difficult to conclude, as the increased body mass in the C-HFS compared to the C-CHOW group rats requires more force and would presumably be associated with a greater mechanical load in C-HFS rats^90^. Our finding, thus, may indicate a reduced ability to adapt to external stresses in the medial gastrocnemius of C-HFS rats^46,70,72^.

Despite muscle quality (i.e., Force/Muscle Mass) being unaltered in the C-HFS rats, indirect evidence suggests that there were significant differences in muscle architecture between C-HFS and C-CHOW group rats. The shift to shorter muscle length in the passive and active FLR of the medial gastrocnemius of the C-HFS rats suggests that the number of sarcomeres-in-series^91^ were lower in C-HFS compared to C-CHOW group rats. Therefore, despite maximal isometric muscle force being maintained, force at a given absolute shortening velocity and the maximal unloaded shortening velocity were likely compromised in C-HFS rats^92,93^. A decrease in serial sarcomeres^94^ would be associated with greater sarcomere excursion for a given change in joint angle^95,96^, and therefore greater sarcomere passive force^97–100^, which would likely be reflected at the whole muscle level. With sarcomere excursion considered a primary regulator of the number of serial sarcomeres^95^, the present findings suggest that C-HFS rats experienced smaller muscle length changes, and therefore may utilize a reduced joint range of motion compared to C-CHOW rats. However, with the mechanical properties of isolated muscles not always reflecting the functional demands of in vivo synergistic groups of muscles in an obvious way^101–103^, one can only speculate on how the changes in medial gastrocnemius mechanics would be manifested when assessing movement patterns of C-HFS rats.

There is evidence of a reduction in oxidative enzyme activity and an increase in lipid content in skeletal muscle in obesity in the three major fiber types^14^. However, the impact of obesity is thought to differ in muscles with different proportions of muscle fiber types^13^. The gastrocnemius and vastus lateralis in Sprague–Dawley rats are primarily fast-twitch fibred muscles, while the soleus is almost exclusively slow-twitch fibred^104^. Previous studies in rodents have shown that after exposure to a high-fat diet, intramuscular fat accumulation is more significant in fast-twitch muscles than in slow-twitch muscles^56,57^. It seems that the high oxidative capacity of slow-twitch muscles may be protective against obesity-induced muscle damage^57^, while the high glycolytic capacity in muscles, such as the vastus lateralis, leads more readily to obesity-induced fat accumulation, inflammation and fibrosis^16^.

Moreover, early and potentially deleterious changes in muscle, such as fibrosis and local inflammation, have been reported following exposure to a high-fat-sucrose diet in the vastus lateralis^16^, which has a similar mixed fiber type composition as the gastrocnemius^57^. The high fast-twitch fibre composition of the gastrocnemius and its low aerobic capacity compared to the rat soleus, might help explain the mechanical changes in force observed in our experiments. However, further studies that systematically test the hypothesis that aerobic capacity of a muscle (i.e., high slow-twitch fibre content) protects against diet- and obesity-induced muscle damage need to be performed before strong conclusions are warranted.

## Conclusion

We conclude from the results of this study that the early progression of metabolic syndrome in very young male rats leads to impairments in muscle remodeling, consistent with the literature^45,72,89^. In addition, development of obesity and increased body weight in such young animals may have led to altered movement patterns^105–107^, specifically reduced hind-limb joint excursion, which may explain the shorter muscle fibers in MG muscle of C-HFS compared to C-CHOW group rats^95,108,109^. Shortened muscle fibers have also been detected in pediatric obese populations^24^. Shorter muscle fibers have been associated with a decreased metabolic cost of force production^110^, as a smaller muscle volume is activated for a given force output^92,111^. Whether a reduction in the energetic cost of movement is the intent when muscle fibers shorten with obesity or is merely a byproduct of altered movement patterns should be addressed in future studies. These studies provide new understanding regarding the impact of diet on muscles during active growth and maturation.

## Supplementary Information

Supplementary Information.

## Data Availability

All relevant data generated or analysed during this study are included in this published article and its Supplementary Information file.

## References

[1] Busutil R (2017). The impact of obesity on health-related quality of life in Spain. Health Qual. Life Outcomes.

[2] Hales, C. M., Carroll, M. D., Fryar, C. D. & Ogden, C. L. Prevalence of obesity among adults and youth: United States, 2015–2016. *NCHS Data Brief* 1–8 (2017). 29155689

[3] Hurst J, James RS, Cox VM, Hill C, Tallis J (2019). Investigating a dose-response relationship between high-fat diet consumption and the contractile performance of isolated mouse soleus, EDL and diaphragm muscles. Eur. J. Appl. Physiol..

[4] Canadian Obesity Network. *Obesity in Canada - Obesity Canada* (2016).

[5] Rauch R (2012). Muscle force and power in obese and overweight children. J. Musculoskelet. Neuronal Interact..

[6] DiFeliceantonio AG (2018). Supra-additive effects of combining fat and carbohydrate on food reward. Cell Metab..

[7] Nguyen DM, El-Serag HB (2010). The epidemiology of obesity. Gastroenterol. Clin. North Am..

[8] Small DM, DiFeliceantonio AG (2019). Processed foods and food reward. Science.

[9] Teasdale N (2013). Obesity alters balance and movement control. Curr. Obes. Rep..

[10] Houmard JA, Pories WJ, Dohm GL (2011). Is there a metabolic program in the skeletal muscle of obese individuals?. J. Obes..

[11] Hulver MW (2003). Skeletal muscle lipid metabolism with obesity. Am. J. Physiol.-Endocrinol. Metab..

[12] Prentki M, Madiraju SRM (2008). Glycerolipid metabolism and signaling in health and disease. Endocr. Rev..

[13] He J, Watkins S, Kelley DE (2001). Skeletal muscle lipid content and oxidative enzyme activity in relation to muscle fiber type in type 2 diabetes and obesity. Diabetes.

[14] Komiya Y (2017). Mouse soleus (slow) muscle shows greater intramyocellular lipid droplet accumulation than EDL (fast) muscle: Fiber type-specific analysis. J. Muscle Res. Cell Motil..

[15] Stuart CA (2013). Slow-twitch fiber proportion in skeletal muscle correlates with insulin responsiveness. J. Clin. Endocrinol. Metab..

[16] Collins KH (2016). High-fat high-sucrose diet leads to dynamic structural and inflammatory alterations in the rat vastus lateralis muscle. J. Orthop. Res..

[17] Goodpaster BH, Wolf D (2004). Skeletal muscle lipid accumulation in obesity, insulin resistance, and type 2 diabetes. Pediatr. Diabetes.

[18] Hilton TN, Tuttle LJ, Bohnert KL, Mueller MJ, Sinacore DR (2008). Excessive adipose tissue infiltration in skeletal muscle in individuals with obesity, diabetes mellitus, and peripheral neuropathy: Association with performance and function. Phys. Ther..

[19] Prado CM (2008). Prevalence and clinical implications of sarcopenic obesity in patients with solid tumours of the respiratory and gastrointestinal tracts: A population-based study. Lancet Oncol..

[20] Tallis J, James RS, Seebacher F (2018). The effects of obesity on skeletal muscle contractile function. J. Exp. Biol..

[21] Abdelmoula A (2012). Knee extension strength in obese and nonobese male adolescents. Appl. Physiol. Nutr. Metab..

[22] Garcia-Vicencio S (2016). The bigger, the stronger? Insights from muscle architecture and nervous characteristics in obese adolescent girls. Int. J. Obes..

[23] Maffiuletti NA (2007). Differences in quadriceps muscle strength and fatigue between lean and obese subjects. Eur. J. Appl. Physiol..

[24] Maffiuletti NA, Jubeau M, Agosti F, De Col A, Sartorio A (2008). Quadriceps muscle function characteristics in severely obese and nonobese adolescents. Eur. J. Appl. Physiol..

[25] Blimkie CJR, Sale DG, Bar-Or O (1990). Voluntary strength, evoked twitch contractile properties and motor unit activation of knee extensors in obese and non-obese adolescent males. Eur. J. Appl. Physiol..

[26] Bray GA (2018). The science of obesity management: An endocrine society scientific statement. Endocr. Rev..

[27] Bott KN (2017). Musculoskeletal structure and function in response to the combined effect of an obesogenic diet and age in male C57BL/6J mice. Mol. Nutr. Food Res..

[28] Ciapaite J (2015). Fiber-type-specific sensitivities and phenotypic adaptations to dietary fat overload differentially impact fast- versus slow-twitch muscle contractile function in C57BL/6J mice. J. Nutr. Biochem..

[29] Hill C, James R, Cox V, Tallis J (2019). Does dietary-induced obesity in old age impair the contractile performance of isolated mouse soleus, extensor digitorum longus and diaphragm skeletal muscles?. Nutrients.

[30] Shortreed KE (2009). Muscle-specific adaptations, impaired oxidative capacity and maintenance of contractile function characterize diet-induced obese mouse skeletal muscle. PLoS ONE.

[31] Tallis J, Hill C, James RS, Cox VM, Seebacher F (2017). The effect of obesity on the contractile performance of isolated mouse soleus, EDL, and diaphragm muscles. J. Appl. Physiol..

[32] Thomas MM (2014). Early oxidative shifts in mouse skeletal muscle morphology with high-fat diet consumption do not lead to functional improvements. Physiol. Rep..

[33] Eshima H (2017). Long-term, but not short-term high-fat diet induces fiber composition changes and impaired contractile force in mouse fast-twitch skeletal muscle. Physiol. Rep..

[34] Matsakas A (2015). Investigating mechanisms underpinning the detrimental impact of a high-fat diet in the developing and adult hypermuscular myostatin null mouse. Skeletal Muscle.

[35] Kennedy AJ, Ellacott KLJ, King VL, Hasty AH (2010). Mouse models of the metabolic syndrome. Dis. Model. Mech..

[36] Long Z (2017). Evolution of metabolic disorder in rats fed high sucrose or high fat diet: Focus on redox state and mitochondrial function. Gen. Comp. Endocrinol..

[37] Russell JC, Proctor SD (2006). Small animal models of cardiovascular disease: Tools for the study of the roles of metabolic syndrome, dyslipidemia, and atherosclerosis. Cardiovasc. Pathol..

[38] Woodie L, Blythe S (2018). The differential effects of high-fat and high-fructose diets on physiology and behavior in male rats. Nutr. Neurosci..

[39] Eckel RH, Grundy SM, Zimmet PZ (2005). The metabolic syndrome. The Lancet.

[40] Weiss R, Bremer AA, Lustig RH (2013). What is metabolic syndrome, and why are children getting it?. Ann. N. Y. Acad. Sci..

[41] Frayn K (2002). Adipose tissue as a buffer for daily lipid flux. Diabetologia.

[42] Ishino S (2017). Glucose uptake of the muscle and adipose tissues in diabetes and obesity disease models: Evaluation of insulin and β3-adrenergic receptor agonist effects by 18F-FDG. Ann. Nucl. Med..

[43] Semple RK (2009). Postreceptor insulin resistance contributes to human dyslipidemia and hepatic steatosis. J. Clin. Investig..

[44] Stern JH, Rutkowski JM, Scherer PE (2016). Adiponectin, leptin, and fatty acids in the maintenance of metabolic homeostasis through adipose tissue crosstalk. Cell Metab..

[45] Valentine RJ, Coughlan KA, Ruderman NB, Saha AK (2014). Insulin inhibits AMPK activity and phosphorylates AMPK Ser485/491 through Akt in hepatocytes, myotubes and incubated rat skeletal muscle. Arch. Biochem. Biophys..

[46] Akhmedov D, Berdeaux R (2013). The effects of obesity on skeletal muscle regeneration. Front. Physiol..

[47] D’Souza DM (2015). Diet-induced obesity impairs muscle satellite cell activation and muscle repair through alterations in hepatocyte growth factor signaling. Physiol. Rep..

[48] Tomlinson DJ, Erskine RM, Winwood K, Morse CI, Onambélé GL (2014). The impact of obesity on skeletal muscle architecture in untrained young vs. old women. J. Anat..

[49] Harris RBS, Apolzan JW (2012). Changes in glucose tolerance and leptin responsiveness of rats offered a choice of lard, sucrose, and chow. Am. J. Physiol.-Regul. Integr. Comp. Physiol..

[50] Yang Z-H, Miyahara H, Takeo J, Katayama M (2012). Diet high in fat and sucrose induces rapid onset of obesity-related metabolic syndrome partly through rapid response of genes involved in lipogenesis, insulin signalling and inflammation in mice. Diabetol. Metab. Syndr..

[51] Louer CR (2012). Diet-induced obesity significantly increases the severity of posttraumatic arthritis in mice. Arthritis Rheum..

[52] Eller LK, Reimer RA (2010). Dairy protein attenuates weight gain in obese rats better than whey or casein alone. Obesity.

[53] Triantaphyllidou I-E (2013). Perturbations in the HDL metabolic pathway predispose to the development of osteoarthritis in mice following long-term exposure to western-type diet. Osteoarthr. Cartilage.

[54] Rios JL (2019). Protective effect of prebiotic and exercise intervention on knee health in a rat model of diet-induced obesity. Sci. Rep..

[55] Collins KH, Reimer RA, Seerattan RA, Leonard TR, Herzog W (2015). Using diet-induced obesity to understand a metabolic subtype of osteoarthritis in rats. Osteoarthr. Cartilage.

[56] Collins KH (2016). A high-fat high-sucrose diet rapidly alters muscle integrity, inflammation and gut microbiota in male rats. Sci. Rep..

[57] Collins KH (2017). Acute and chronic changes in rat soleus muscle after high-fat high-sucrose diet. Physiol. Rep..

[58] Collins KH (2020). Impact of age on host responses to diet-induced obesity: Development of joint damage and metabolic set points. J. Sport Health Sci..

[59] Kilkenny C, Browne WJ, Cuthill IC, Emerson M, Altman DG (2010). Improving bioscience research reporting: The ARRIVE guidelines for reporting animal research. PLoS Biol..

[60] NoyeTuplin EW (2022). Dietary fiber combinations to mitigate the metabolic, microbial, and cognitive imbalances resulting from diet-induced obesity in rats. FASEB J..

[61] Matsuda M, DeFronzo RA (1999). Insulin sensitivity indices obtained from oral glucose tolerance testing: Comparison with the euglycemic insulin clamp. Diabetes Care.

[62] Reno C, Marchuk L, Sciore P, Frank CB, Hart DA (1997). Rapid isolation of total RNA from small samples of hypocellular, dense connective tissues. BioTechniques.

[63] Herzog W, Leonard TR (1997). Depression of cat soleus forces following isokinetic shortening. J. Biomech..

[64] Rode C, Siebert T, Herzog W, Blickhan R (2009). The effects of parallel and series elastic components on the active cat soleus force-length relationship. J. Mech. Med. Biol..

[65] de Brito Fontana H, Herzog W (2016). Vastus lateralis maximum force-generating potential occurs at optimal fascicle length regardless of activation level. Eur. J. Appl. Physiol..

[66] Gavrilova O (1999). Torpor in mice is induced by both leptin-dependent and -independent mechanisms. Proc. Natl. Acad. Sci. USA.

[67] Rosenbaum M (2018). Triiodothyronine and leptin repletion in humans similarly reverse weight-loss-induced changes in skeletal muscle. Am. J. Physiol.-Endocrinol. Metab..

[68] Reilly SM, Saltiel AR (2017). Adapting to obesity with adipose tissue inflammation. Nat. Rev. Endocrinol..

[69] Sørensen TIA, Virtue S, Vidal-Puig A (2010). Obesity as a clinical and public health problem: Is there a need for a new definition based on lipotoxicity effects?. Biochim. Biophys. Acta.

[70] Brown LA (2015). Diet-induced obesity alters anabolic signalling in mice at the onset of skeletal muscle regeneration. Acta Physiol..

[71] Musarò A (2014). The basis of muscle regeneration. Adv. Biol..

[72] Tamilarasan KP (2012). Skeletal muscle damage and impaired regeneration due to LPL-mediated lipotoxicity. Cell Death Dis..

[73] Virtue S, Vidal-Puig A (2010). Adipose tissue expandability, lipotoxicity and the metabolic syndrome: An allostatic perspective. Biochim. Biophys. Acta (BBA).

[74] Halaas JL (1995). Weight-reducing effects of the plasma protein encoded by the obese gene. Science.

[75] Shanik MH (2008). Insulin resistance and hyperinsulinemia. Diabetes Care.

[76] Vega GL, Barlow CE, Grundy SM, Leonard D, DeFina LF (2014). Triglyceride–to–high-density-lipoprotein-cholesterol ratio is an index of heart disease mortality and of incidence of type 2 diabetes mellitus in men. J. Investig. Med..

[77] Iozzo P (2009). Viewpoints on the way to the consensus session. Diabetes Care.

[78] Hotamisligil GS (2006). Inflammation and metabolic disorders. Nature.

[79] Borén J, Taskinen M-R, Olofsson S-O, Levin M (2013). Ectopic lipid storage and insulin resistance: A harmful relationship. J. Intern. Med..

[80] Puri V, Czech MP (2008). Lipid droplets: FSP27 knockout enhances their sizzle. J. Clin. Investig..

[81] Lee B, Shao J (2012). Adiponectin and lipid metabolism in skeletal muscle. Acta Pharm. Sin. B.

[82] Fu X (2016). Obesity impairs skeletal muscle regeneration through inhibition of AMPK. Diabetes.

[83] Dinarello CA (2011). Interleukin-1 in the pathogenesis and treatment of inflammatory diseases. Blood.

[84] Dinarello CA (2011). A clinical perspective of IL-1β as the gatekeeper of inflammation. Eur. J. Immunol..

[85] Madej MP, Töpfer E, Boraschi D, Italiani P (2017). Different regulation of interleukin-1 production and activity in monocytes and macrophages: Innate memory as an endogenous mechanism of IL-1 inhibition. Front. Pharmacol..

[86] McIlwain DR, Berger T, Mak TW (2013). Caspase functions in cell death and disease. Cold Spring Harb. Perspect. Biol..

[87] Kam PCA, Ferch NI (2000). Apoptosis: Mechanisms and clinical implications. Anaesthesia.

[88] Karalaki M, Fili S, Philippou A, Koutsilieris M (2009). Muscle regeneration: Cellular and molecular events. In Vivo.

[89] Wynn TA (2007). Common and unique mechanisms regulate fibrosis in various fibroproliferative diseases. J. Clin. Investig..

[90] Hodson-Tole EF, Wakeling JM (2010). The influence of strain and activation on the locomotor function of rat ankle extensor muscles. J. Exp. Biol..

[91] Zuurbier CJ, Huijing PA (1993). Changes in geometry of activily shortening unipennate rat gastrocnemius muscle. J. Morphol..

[92] Lieber RL, Ward SR (2011). Skeletal muscle design to meet functional demands. Philos. Trans. R. Soc. B.

[93] Narici M, Franchi M, Maganaris C (2016). Muscle structural assembly and functional consequences. J. Exp. Biol..

[94] Williams PE, Goldspink G (1978). Changes in sarcomere length and physiological properties in immobilized muscle. J. Anat..

[95] Koh TJ, Herzog W (1998). Excursion is important in regulating sarcomere number in the growing rabbit tibialis anterior. J. Physiol..

[96] Rassier DE, MacIntosh BR, Herzog W (1999). Length dependence of active force production in skeletal muscle. J. Appl. Physiol..

[97] Herzog JA, Leonard TR, Jinha A, Herzog W (2012). Are titin properties reflected in single myofibrils?. J. Biomech..

[98] Leonard TR, DuVall M, Herzog W (2010). Force enhancement following stretch in a single sarcomere. Am. J. Physiol. Cell Physiol..

[99] Leonard TR, Herzog W (2010). Regulation of muscle force in the absence of actin-myosin-based cross-bridge interaction. Am. J. Physiol. Cell Physiol..

[100] Matthiasdottir S, Hahn M, Yaraskavitch M, Herzog W (2014). Muscle and fascicle excursion in children with cerebral palsy. Clin. Biomech..

[101] Herzog W (1992). Force-length properties and functional demands of cat gastrocnemius, soleus and plantaris muscles. J. Biomech..

[102] de Brito Fontana H, Han SW, Sawatsky A, Herzog W (2018). The mechanics of agonistic muscles. J. Biomech..

[103] de Brito Fontana H, de Campos D, Sawatsky A, Han S, Herzog W (2019). Why do muscles lose torque potential when activated within their agonistic group?. J. Exp. Biol..

[104] Rakus D, Gizak A, Deshmukh A, Wiśniewski JR (2015). Absolute quantitative profiling of the key metabolic pathways in slow and fast skeletal muscle. J. Proteome Res..

[105] Colné P, Frelut ML, Pérès G, Thoumie P (2008). Postural control in obese adolescents assessed by limits of stability and gait initiation. Gait Posture.

[106] Nantel J, Brochu M, Prince F (2006). Locomotor strategies in obese and non-obese children*. Obesity.

[107] Strutzenberger G, Richter A, Schneider M, Mündermann A, Schwameder H (2011). Effects of obesity on the biomechanics of stair-walking in children. Gait Posture.

[108] Goldspink G (1985). Malleability of the motor system: A comparative approach. J. Exp. Biol..

[109] Herring SW, Grimm AF, Grimm BR (1984). Regulation of sarcomere number in skeletal muscle: A comparison of hypotheses. Muscle Nerve.

[110] Biewener AA (2016). Locomotion as an emergent property of muscle contractile dynamics. J. Exp. Biol..

[111] Fletcher JR, MacIntosh BR (2017). Running economy from a muscle energetics perspective. Front. Physiol..

