# Supplementary material for: Alterations in skeletal muscle morphology and mechanics in juvenile male Sprague Dawley rats exposed to a high-fat high-sucrose diet
Source: Sci Rep. 2023 Jul 25;13:12013. doi: 10.1038/s41598-023-38487-x (PMC10368627; doi:10.1038/s41598-023-38487-x)
Supplement: Supplementary file 1 — Supplementary Information. [file 41598_2023_38487_MOESM1_ESM.pdf]

Alterations in skeletal muscle morphology and mechanics in juvenile male Sprague Dawley rats exposed to a high-fat high-sucrose diet

Mauricio Delgado-Bravo[1,5], David A. Hart[1,2,4], Raylene A. Reimer[1,3], Walter Herzog[1,2,*]

Supplementary Material- Table 1.
Sequences and origin for rat primers used for real-time qPCR procedures to assess medial gastrocnemius tissue.

Gene	Forward	Reverse	Origin
**18S**	TGG TCG CTC GCT CCT CTC C	CGC CTG CTG CCT TCC TTG G	NR_003286
**Fsp27**	TGG GTC AGA GAA ACA ATG GAG T	AGT GCT CAC TGC TAC ATG CC	NM_001024333
**Adiponectin**	CAC TCA GCA TTC AGC GTA GG	GCC AGT GCT GCC GTC ATA AT	NM_144744
**Leptin**	CCT GTG GCT TTG GTC CTA TCT G	CTG CTC AAA GCC ACC ACC TCT G	NM_013076
**MCP-1**	ACT ATG CAG GTC TCT GTC AC	TGC CAG TGA ATG AGT AGC AG	M54771
**IL-6**	TCA CAG AAG GAG TGG CTA AG	ACC ACA GTG AGG AAT GTC CA	NM_012589
**TNFα**	CAC GCT CTT CTG TCT ACT GA	GGC CAT GGA ACT GAT GAG AG	X66539
**IL-1β**	AAC CTG CTG GTG TGT GAC GTT C	CAG CAC GAG GCT TTT TTG TTG T	NM_008361
**Cytochrome-C**	CTT GGG CTA GAG AGC GGG A	GCT TGC CTC CTT TTT CCA CAG	NM_012839
**Caspase-3**	GGA GCT TGG AAC GCG AAG AA	ACA CAA GCC CAT TTC AGG GT	NM_012922
**Pax7**	TGC CCT CAG TGA GTT CGA TT	GGA GGT CGG GTT CTG ATT CC	NM_001191984
**MyoD**	CAA GCG CAA GAC CAC TAA CG	TTC AAT GTA GCG GAT GGC GT	NM_176079
**Myf5**	ACG TCC CCA ATG AGA TTA GCA	GGG CTT CAC TTA CTG GGC AT	NM_001106783
**Myogenin**	AGA AGC GCA GGC TCA AGA AA	GCC TGT AGG CGC TCA ATG TA	M24393
**MRF4**	GCC CCT TTC CGC CTA ATC AT	TAA AAG CCC CAA GCC GAA GG	M27151

Supplementary Material- Table 2. Dynamic blood insulin and glucose test (0 to 120 minutes).

		Insulin (AUC)						Glucose (AUC)					
		**Pre**	**15**	**30**	**60**	**120**	**Total**	**Pre**	**15**	**30**	**60**	**120**	**Total**
**Chow**	**Mean**	77.5	2560.6	3171.4	5379.4	9035.8	20224.6	2.9	106.8	160.4	353.6	663.0	1286.7
	**SD**	42.0	1254.3	1546.6	2550.1	6116.3	6917.7	0.4	9.9	13.6	58.5	149.8	210.9
**HFS**	**Mean**	95.5	4493.2	6365.8	11915.7	20250.8	36996.1	3.1	120.0	169.2	378.6	799.1	1470.0
	**SD**	37.9	2863.7	3969.2	6065.3	10824.5	12180.9	0.5	26.2	34.6	64.3	126.5	243.1

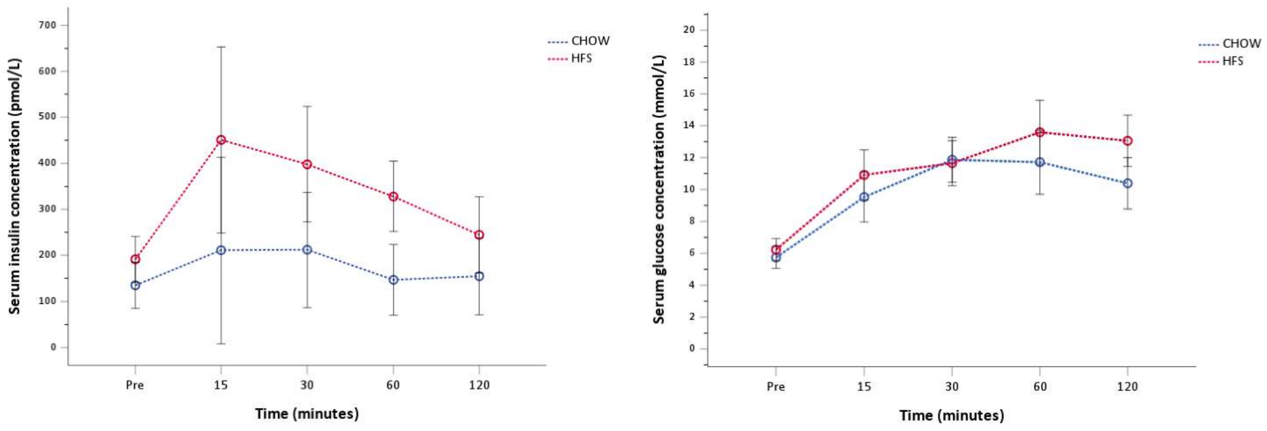

Supplementary Material- Figure 1. Dynamic blood insulin and glucose test (0 to 120 minutes). For insulin test Time effect and Diet effect was significant ($p < .05$). For glucose test the main effect of Time was significant ($p < .05$). Data points represent the average, with error bars representing the 95% interval confidence.
